# Folding Free Energies of 5′-UTRs Impact Post-Transcriptional Regulation on a Genomic Scale in Yeast

**DOI:** 10.1371/journal.pcbi.0010072

**Published:** 2005-12-09

**Authors:** Markus Ringnér, Morten Krogh

**Affiliations:** Complex Systems Division, Department of Theoretical Physics, Lund University, Lund, Sweden; Washington University in St. Louis, United States of America

## Abstract

Using high-throughput technologies, abundances and other features of genes and proteins have been measured on a genome-wide scale in *Saccharomyces cerevisiae*. In contrast, secondary structure in 5′–untranslated regions (UTRs) of mRNA has only been investigated for a limited number of genes. Here, the aim is to study genome-wide regulatory effects of mRNA 5′-UTR folding free energies. We performed computations of secondary structures in 5′-UTRs and their folding free energies for all verified genes in *S. cerevisiae*. We found significant correlations between folding free energies of 5′-UTRs and various transcript features measured in genome-wide studies of yeast. In particular, mRNAs with weakly folded 5′-UTRs have higher translation rates, higher abundances of the corresponding proteins, longer half-lives, and higher numbers of transcripts, and are upregulated after heat shock. Furthermore, 5′-UTRs have significantly higher folding free energies than other genomic regions and randomized sequences. We also found a positive correlation between transcript half-life and ribosome occupancy that is more pronounced for short-lived transcripts, which supports a picture of competition between translation and degradation. Among the genes with strongly folded 5′-UTRs, there is a huge overrepresentation of uncharacterized open reading frames. Based on our analysis, we conclude that (i) there is a widespread bias for 5′-UTRs to be weakly folded, (ii) folding free energies of 5′-UTRs are correlated with mRNA translation and turnover on a genomic scale, and (iii) transcripts with strongly folded 5′-UTRs are often rare and hard to find experimentally.

## Synopsis

In cells, proteins are made from messenger RNA copied from genes in the DNA. The amount of each protein needs to be controlled by cells. For this purpose, cells use a strategy that includes decomposing RNA and varying the number of proteins made from each RNA. One part of the RNA molecule is called the 5′–untranslated region (UTR), and it is known that this region can fold into a three-dimensional structure. For some genes, such structures are important for protein production. In this article, structures in 5′-UTRs are calculated for all genes in the yeast *Saccharomyces cerevisiae*. The authors show that structures in 5′-UTRs likely play a role in RNA decomposition and protein production for many genes in the genome: RNA molecules with weakly folded 5′-UTRs live relatively longer and produce more proteins. This study provides an example of how genome-wide computational analysis complements experimental results.

## Introduction

Regulation of gene expression is important for many cellular processes. Numerous studies have focused on the transcriptional level to investigate under what conditions a gene is transcribed and to what extent. These investigations have led to descriptions of system architectures, in which the activity of specific transcription factors regulates the activity of downstream target genes in such a way that the combined activity results in large developmental or physiological programs. In recent years, such descriptions have benefited from DNA microarray technology, which has provided overall mRNA levels for many systems. However, much less is known about the system architecture of regulation of gene expression at the post-transcriptional level, including regulation of mRNA subcellular localization, stability, and translation rate.

**Figure 1:**
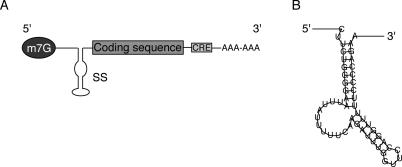
Structure of Yeast mRNA (A) The mRNA has a tripartite structure consisting of a 5′-UTR, a coding region, and a 3′-UTR. CRE, *cis*-acting regulatory element; m7G, 7-methyl-guanosine cap; SS, secondary structure. (B) The computed minimum free energy secondary structure for the 5′-UTR of the gene YBR296C-A.

mRNA consists of three parts: a 5′–untranslated region (UTR) beginning with a 7-methyl-guanosine cap, a coding region, and a 3′-UTR ending in a poly(A) tail (Figure 1A). UTRs of mRNAs are known to be a crucial part of post-transcriptional regulation [1]. In yeast, the exact lengths of 5′- and 3′-UTRs are unknown for most genes. Mignone et al. [1] estimated the average lengths for yeast as 134 nucleotides (nt) for 5′-UTRs and 237 nt for 3′-UTRs. Later, Hurowitz and Brown [2] performed genome-wide measurements of total transcript lengths and calculated the average combined 5′- and 3′-UTR length to be 260 nt. *Cis*-acting sequence motifs in 3′-UTRs can interact with specific RNA-binding proteins (RBPs) to direct subcellular localization [3] and stability [4] of mRNAs. DNA microarrays have also enabled a growing body of work on global analysis of RBPs that supports the importance of RBPs in many cellular processes through post-transcriptional regulation of mRNAs [5–7]. Translation of the majority of mRNAs depends on cap-dependent ribosomal scanning of 5′-UTRs [8], and this process is influenced by features of 5′-UTRs. For example, ribosomal scanning is severely hampered by 5′-UTRs containing start codons or secondary structure [9–15].

The purpose of mRNA degradation is 2-fold: to regulate transcript abundance and to destroy faulty transcripts. Degradation of mRNA in yeast occurs via 5′ to 3′ exonucleotic, 3′ to 5′ exonucleotic, and endonucleotic pathways [16–19]. Regulation of transcript abundance via the exonucleotic pathways occurs by first shortening the poly(A) tail followed either by removal of the 5′ cap, resulting in rapid 5′ to 3′ degradation, or by degradation from the 3′ end without prior decapping [18,20]. The dual importance of the cap structure, for translation initiation and 5′ to 3′ mRNA decay, has led to the hypothesis that there is a competition between translation and decay for access to the cap [13,20,21]. Transcripts with 5′-UTRs that hamper their translation often encode for proteins that need to be strongly and finely regulated, such as growth factors, transcription factors, and proto-oncogenes [14], suggesting that 5′-UTRs are sometimes structured in a way to prevent harmful overproduction of regulatory proteins. Indeed, some diseases are caused by mutations in 5′-UTRs [22,23]. In agreement with this picture, proteins involved in the regulation of dynamic cellular processes such as transcription, signal transduction, cell cycle control, and metabolism have long UTRs [2].

To our knowledge no one has found genome-wide associations between secondary structure in 5′-UTRs and mRNA half-life, translation rates, or other transcript features. For example, Bernstein et al. [24] performed a genome-wide experiment of mRNA decay in *Escherichia coli* and found no association to secondary structure in UTRs. To study the regulatory effects of secondary structure in UTRs, we performed genome-wide computations of secondary structures and their folding free energies in 5′-UTRs for 5,888 verified genes in *Saccharomyces cerevisiae*. The folding free energy is the difference in free energy between the unfolded and folded state. For a given mRNA length, a lower folding free energy corresponds to a more stable secondary structure. We analyzed associations between folding free energies and various transcript features including translation and decay rates. One result of our analysis is that low folding free energy of 5′-UTRs is, on average, associated with low translation rates and high transcript turnover, in concordance with previous results for single genes (e.g., [13]). We also found that 5′-UTRs on average are more weakly folded than random sequences with the same dinucleotide frequencies, and than intergenic, coding, and 3′-UTR sequences. Strikingly, genes with unknown function were enriched among genes with strongly folded 5′-UTRs.

## Results

### Folding Free Energies of 5′-UTRs

To investigate secondary structure in 5′-UTRs, we used the Vienna RNA package [25] to compute secondary structures and the corresponding free energy changes for folding (Δ*G*). The lower Δ*G* is, the more strongly folded is the secondary structure. Using 5′-UTRs of length 50 nt, the average Δ*G* was −4.3 kcal/mol (standard deviation [SD] = 2.9 kcal/mol) for the 5,888 open reading frames (ORFs) investigated. The range of Δ*G* was from −18.1 kcal/mol to 0 kcal/mol. The lowest value of Δ*G,* −18.1 kcal/mol, was obtained for the gene YBR296C-A, whose computed 5′-UTR secondary structure is illustrated in Figure 1B. There were 231 5′-UTRs with folding free energies below −10 kcal/mol. These thermodynamically most stable structures had on average 12.9 base pairs (SD = 2.2), i.e., more than half of the bases were typically paired. Their average GC-content was 47% (SD = 7%). The structures were mostly hairpins similar to Figure 1B with unpaired bases in internal or bulge loops or at the ends of the sequences, but also structures containing two hairpins were found. There were 727 5′-UTRs with folding energies above −1 kcal/mol. These 5′-UTRs formed minimum free energy structures having on average 2.6 base pairs (SD = 3.0) and their average GC-content was 29% (SD = 7%).

### Folding Free Energies of Other Genomic Regions

**Figure 2:**
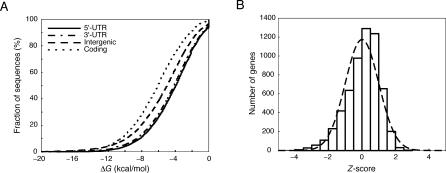
Folding Free Energies of 5′-UTRs (A) Cumulative distributions of folding free energies, Δ*G,* are shown for 5,888 ORFs for 5′-UTRs (50 nt upstream of the ORF; solid line), 3′-UTRs (50 nt downstream of the ORF; dashed-dotted line), coding sequences (50-nt sequences following downstream of the start codon of each ORF; dotted line), and 5,888 sequences of length 50 nt selected randomly from intergenic regions (dashed line). (B) Distribution of *Z*-scores for 5′-UTRs of 5,888 ORFs. Each 5′-UTR sequence was shuffled 100 times and a *Z*-score was calculated for each to compare the folding free energy of the native sequence to the shuffled sequences. A histogram of these *Z*-scores is shown together with a standard normal distribution (dashed line).

Folding free energies were computed for three control groups, all containing 5,888 sequences of length 50 nt. The first group consisted of randomly chosen sequences from intergenic regions and had an average Δ*G* of −5.4 kcal/mol (SD = 3.4 kcal/mol). The second group consisted of the first 50 nt of the 3′-UTR of each ORF and had an average Δ*G* of −4.5 kcal/mol (SD = 3.1 kcal/mol). The third group consisted of the 50 nt located after the start codon of each ORF and had an average Δ*G* of −6.3 kcal/mol (SD = 3.2 kcal/mol). The free energies of the 5′-UTRs were significantly higher than those of the three other groups (3′-UTR: *p <* 3 × 10^−4^, intergenic: *p <* 2 × 10^−70^, coding: *p <* 3 × 10^−253^; Mann–Whitney *U* test). Figure 2A shows cumulative distributions of all free energies for the four groups.

### Folding Free Energies of Randomized Sequences

The free energy of secondary structure in RNA is highly dependent on nucleotide composition. A base pair stacking term that depends on dinucleotides contributes to the free energy. We obtained the free energy contributions for each of the 16 possible dinucleotides from Xia et al. [26]. We calculated the dinucleotide frequencies in the four groups of sequences and used the dinucleotide energy contributions as weights in a weighted average of the dinucleotide frequencies. This calculation gave us a rudimentary measure for the contribution to the free energies coming from dinucleotide composition without actually folding the structures. The weighted dinucleotide composition for the four groups was −1.74 kcal/mol for 3′-UTRs, −1.81 kcal/mol for 5′-UTRs, −1.81 kcal/mol for intergenic sequences, and −1.95 kcal/mol for coding sequences. The dinucleotides with lowest free energy are GC, CC, GG and CG, so GC-content is an even simpler measure for the relative contribution of nucleotide composition to the free energy. The GC-content of the four groups was 31% for 3′-UTRs, 34% for 5′-UTRs, 34% for intergenic sequences, and 40% for coding sequences. Interestingly, the two measures are in perfect agreement.

We checked whether the folding free energies of 5′-UTRs were not only higher than for the other groups of sequences, but also different from what was expected from 5′-UTR dinucleotide composition [27]. For this purpose, we used a dinucleotide shuffling algorithm [28,29]. Native 5′-UTR sequences were shuffled 100 times each, and minimum free energies were calculated for all randomized sequences. The mean free energy of the randomized sequences was −4.4 kcal/mol as compared to −4.3 kcal/mol for the native sequences. *Z*-scores were calculated to compare the folding free energy of each 5′-UTR with the free energies of its randomized sequences. 5′-UTRs with positive *Z*-scores had higher folding free energies than the average of their randomized sequences and are therefore thought to have less secondary structure. We found an overabundance of 5′-UTRs with positive *Z*-scores (Figure 2B). The mean value of the *Z*-scores was 0.050 (standard error of the mean [SEM] = 0.013), which is significantly different from zero (*p <* 10^−4^; *t*-test). Also 58% of the 5,888 ORFs had a positive *Z*-score, which is significantly more than expected by chance (*p <* 3 × 10^−35^).

### Folding Free Energies of 5′-UTRs and Transcript Features

**Figure 3:**
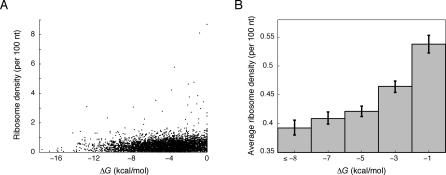
Comparison between Ribosome Densities and Folding Free Energies of 5′-UTRs (A) Scatter plot of mRNA ribosome density and folding free energy of the 5′-UTR (Δ*G*) for 5,888 ORFs. (B) ORFs were grouped based on the change in free energy (Δ*G*). For each energy group, the average ribosome density (±SEM) is shown. From left to right, the number of ORFs in each energy group used to calculate the average density was 573, 796, 1,214, 1,438, and 1,187.

**Figure 4:**
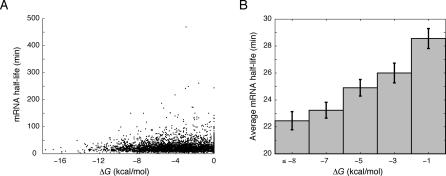
Comparison between mRNA Half-Lives and Folding Free Energies of 5′-UTRs (A) Scatter plot of mRNA half-life and folding free energy of the 5′-UTR (Δ*G*) for 5,888 ORFs. (B) ORFs were grouped based on the folding free energy (Δ*G*). For each energy group, the average mRNA half-life (±SEM) is shown. From left to right, the number of ORFs in each energy group used to calculate the average density was 467, 657, 982, 1,169, and 983.

We investigated the correlation between Δ*G* and the ribosome density measured by Arava et al. [30]. We observed a small but significant correlation (Figure 3). The Pearson correlation was 0.12, with an associated *p*-value of 3 × 10^−16^. Beyer et al. [31] argue that it is preferable to define ribosome density as the number of ribosomes divided by transcript length instead of ORF length. They provide a processed dataset of such ribosome densities, and these densities had a Pearson correlation of 0.09 (*p <* 10^−10^) with Δ*G*. Likewise, using mRNA half-lives measured by Wang et al. [32], we observed a small but significant correlation between Δ*G* and mRNA half-lives (Figure 4). The Pearson correlation was 0.10 (*p <* 3 × 10^−10^). We also found significant correlations between Δ*G* on the one hand and ribosome occupancy, the number of ribosomes bound on the transcript, the mRNA copy number, and protein abundance on the other hand (Table 1). To avoid potential pitfalls in the assumptions used to calculate *p*-values for Pearson correlations, we also calculated Spearman rank correlations. We observed similar results for both correlation measures (Table 1). In contrast to our results for 5′-UTRs, we found no significant correlations between folding free energies of 3′-UTRs and transcript features.

**Table 1:**
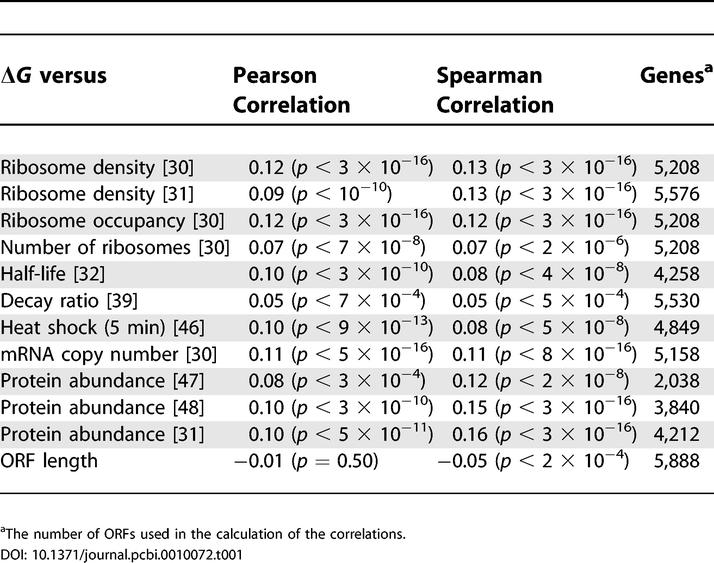
Correlations between Secondary Structure in 5′-UTRs and Transcript Features

As expected, we observed a large correlation between Δ*G* and GC-content for the 5′-UTRs. The Pearson correlation was 0.48 (*p <* 3 × 10^−16^). To rule out that our observed correlations between Δ*G* and transcript features were merely a consequence of GC-content, we investigated whether Δ*G* was correlated with the transcript features independently of GC-content. We regressed the transcript features as a function of GC-content and free energy in a multivariate model. First, significance was calculated for the correlation between GC-content and a transcript feature. Second, significance was calculated for free energy being correlated to the transcript features after subtraction of the GC-content effect. For ribosome density, we obtained *p =* 5 × 10^−4^ for GC-content and *p <* 5 × 10^−14^ for free energy. For mRNA half-life, we obtained *p <* 10^−15^ for GC-content and *p <* 0.004 for free energy. For the combined protein abundance dataset [31], we obtained *p <* 2 × 10^−12^ for GC-content and *p <* 0.0002 for free energy. Similar results were obtained when correcting for weighted dinucleotide composition instead of for GC-content.

### Fast and Slowly Decaying Genes

In order to check whether the relations between various transcript features depended on the half-life of the mRNA, we designated the 1,013 genes with a half-life below 13 min as fast decaying, and the 1,058 genes with a half-life above 33 min as slowly decaying. These cutoffs were chosen to get closest to, and above, 1,000 genes. The only correlations between Δ*G* and any of the other nine transcript features in Table 1 that changed significantly (*p <* 0.001) were with half-life and heat shock: in the fast decaying group of genes, Δ*G* and half-life had a correlation of −0.06, which is significantly different from their correlation of 0.10 among all genes (*p <* 8 × 10^−7^). Similarly in the fast decaying group of genes, Δ*G* and heat shock had a correlation of −0.01, which is significantly different from their correlation of 0.10 among all genes (*p <* 6 × 10^−4^).

### Correlation between Decay and Translation

**Figure 5:**
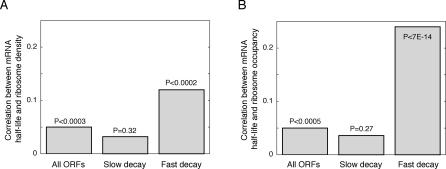
Correlations between Decay and Translation Rates Pearson correlations together with corresponding *p*-values are shown for mRNA half-life versus (A) ribosome density and (B) ribosome occupancy. ORFs were, depending on mRNA half-life, grouped into all ORFs, 1,058 slowly decaying ORFs with *t*_1/2_ ≥ 33 min, and 1,013 fast decaying ORFs with *t*_1/2_ ≤ 13 min.

It has been argued that translational efficiency of a transcript is a determinant of mRNA half-life: decreased translation leads to decreased half-life. Evidence for this model has come from yeast strains either mutated in translation initiation factors [33] or with translation of individual mRNAs inhibited [13]. To see whether such an effect is present globally in yeast without such modifications, we calculated the correlations between half-life on the one hand and ribosome density and ribosome occupancy on the other hand. We found a small, but significant, correlation among all genes. However, for the fast decaying genes the correlations were much stronger, especially between half-life and ribosome occupancy, for which the correlation was 0.24 (Figure 5).

### Gene Ontology Analysis

To see whether folding free energies of 5′-UTRs were associated with functional annotations, we mapped the 5,888 genes to 3,678 Gene Ontology (GO) categories [34]. The genes were ranked according to Δ*G* in both increasing and decreasing order, and a Wilcoxon rank sum test was employed for each GO category [35]. The significant categories, using a very stringent *p*-value cutoff of 10^−8^, corresponding to a Bonferroni corrected cutoff of 4 × 10^−5^, are listed in Table 2. Among genes with strongly folded 5′-UTRs, three categories were significant and no other categories were close to being this significant. Remarkably, these three categories were “molecular function unknown,” “biological process unknown,” and “cellular component unknown.” Among genes with weakly folded 5′-UTRs, 12 categories were significantly overrepresented. Chief among these were categories related to retrotransposons.

**Table 2:**
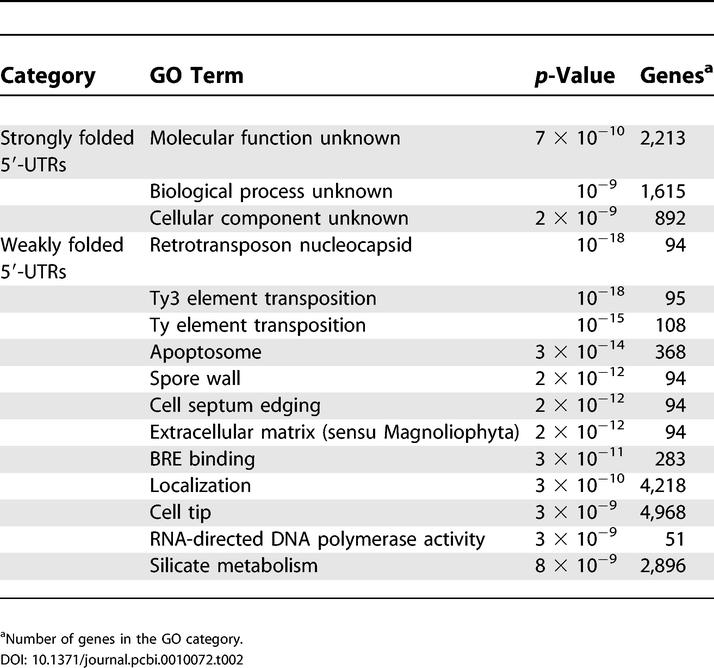
GO Terms Overrepresented among Genes with Strongly and Weakly Folded 5′-UTRs

### RNA-Binding Proteins

Affinity tagging of RBPs followed by microarray hybridizations has been used to obtain genome-wide lists of bound transcripts. We obtained the lists of bound transcripts for the RBPs Yra1, Mex67 [6], and for five members of the Puf family [7]. Furthermore, we identified all the genes whose 3′-UTR contained the consensus motifs for Puf3p, Puf4p, and Puf5p. For each of these ten gene sets, we examined whether there was a significant difference in the number of fast decaying genes relative to slowly decaying genes, and whether there was a significant difference in the number of genes having strongly folded 5′-UTRs relative to genes with weakly folded 5′-UTRs (Table 3). The most significant associations were that transcripts bound by Puf3p, Puf4p, and Puf5p were fast decaying. The Puf3p, Puf4p, and Puf5p motifs confirm this picture. The most significant associations with folding free energy were that Mex67 and Yra1 preferentially bind transcripts with weakly folded 5′-UTRs.

**Table 3:**
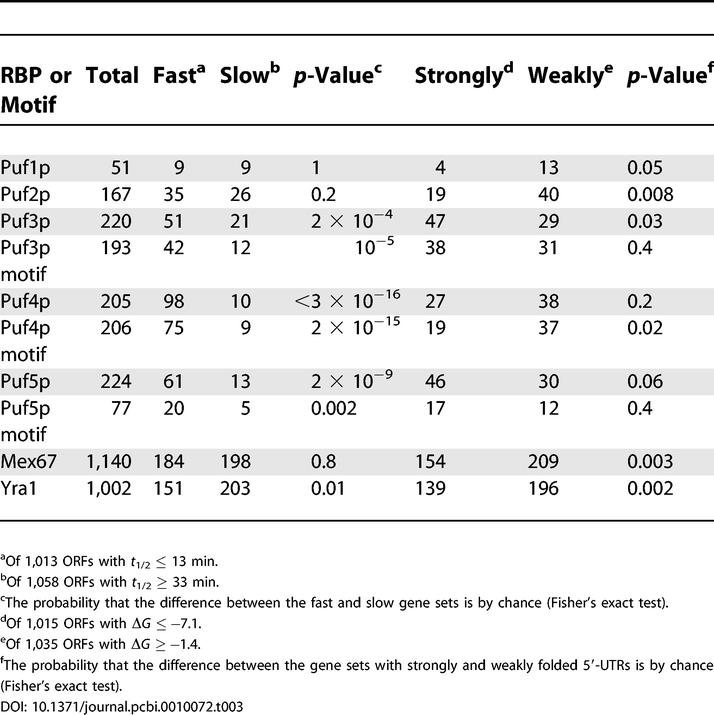
Number of RBP Targets and Sequence Motifs Found in All mRNAs, mRNAs with Fast and Slow Decay Rates, and mRNAs with Strongly and Weakly Folded 5′-UTRs

### Comparison with Longer Upstream Regions

The 5′-UTRs of yeast genes vary in length. In this study, we used the 50 nt upstream of the start codon as a representation of the 5′-UTR. Since 50 nt is shorter than many 5′-UTRs, we also used 100- and 200-nt 5′-UTRs for comparison. The correlation between Δ*G* and ribosome density decreases for longer regions, but is still significant for 100 nt (Table 4). Similar behavior was observed for other transcript features.

**Table 4:**
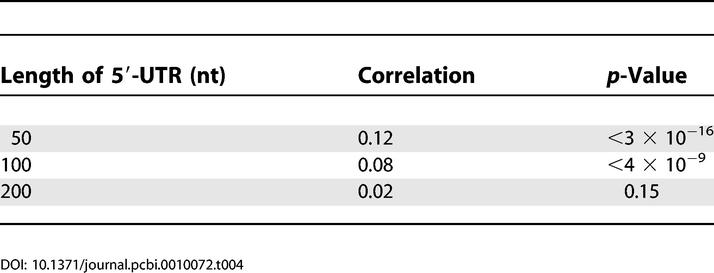
Pearson Correlations between Ribosome Density and Δ*G* in 5′-UTRs of Length 50, 100, and 200 nt

## Discussion

We carried out genome-wide computations of secondary structures in 5′-UTRs of mRNA in yeast, and correlated 5′-UTR folding free energy with various other transcript features. We chose somewhat arbitrarily to fold sequences of length 50 nt upstream of the coding start, because these sequences are almost certainly inside the 5′-UTR. We also folded 100- and 200-nt sequences, and had similar but weaker results (Table 4). Folding of RNA is somewhat local in sequence: when folding 100- or 200-nt upstream sequences, the last 50 nt were typically computed to fold into the same structure as when the 50-nt upstream sequences were folded. Translation has been shown to be most sensitive to secondary structure close to the 5′ end of mRNA [12]. Hence, we think that the weaker results obtained for longer upstream sequences reflect an increase of sequence spanning genomic DNA not being transcribed, and not that secondary structure close to the translation start is most important for the transcript features we have investigated. We used 5′-UTRs of fixed length to avoid comparing free energies for sequences of different lengths. Bernstein et al. [24] used predicted UTRs for each gene in *E. coli* and found no association between secondary structure in UTRs and mRNA half-life. Our different findings may be due to differences between pro- and eukaryotes, or difficulties in comparing UTRs of different length.

To compare 5′-UTRs with other genomic regions, 50-nt sequences from intergenic regions, coding regions, and 3′-UTRs were also folded. These three sets of sequences had significantly lower free energies than the 5′-UTR sequences (see Figure 2A). The folding free energy of RNA depends on both nucleotide composition and the order of the nucleotides. The nucleotide composition, quantified both by GC-content and weighted dinucleotide composition, was similar in 5′-UTRs and intergenic regions, indicating that the difference in free energies between these groups is due to nucleotide order. Indeed, the 5′-UTRs had higher folding free energies than random sequences with the same dinucleotide composition (Figure 2B). In contrast, yeast coding regions have lower folding energies than randomized sequences preserving the encoded protein, the codon usage, and the dinucleotide composition [36]. This opposite behavior is in agreement with the huge difference in folding free energies between coding regions and 5′-UTRs (Figure 2A), even though GC-content probably is more important for this difference. Our results indicate that there has been evolutionary selection for 5′-UTRs to be weakly folded and suggest that folding free energy might be used as one probabilistic component of a gene prediction program.

In line with our observation that 5′-UTRs tend to be weakly folded is our finding that uncharacterized ORFs are overrepresented among the genes with strongly folded 5′-UTRs. Assuming that uncharacterized genes typically are expressed at low levels or under rare conditions, or even are pseudogenes, this finding hints at a larger selective pressure for absence of secondary structure for commonly or highly expressed genes. Confirming this picture is our finding that 5′-UTR folding free energy is significantly positively correlated with mRNA copy number and protein abundance (see Table 1). Since we only investigated verified genes, we could look into the source of the verification of the genes with strongly folded 5′-UTRs. The 5′-UTR of the gene YBR296C-A (see Figure 1B) has the secondary structure with the lowest free energy of all genes, and is annotated as unknown in GO. Remarkably, this gene has only one literature reference, in which Kumar et al. [37] describe an approach for finding overlooked genes in yeast. Of the 137 new genes reported by Kumar et al., 41 are annotated as verified in the *Saccharomyces* Genome Database (SGD). Ten of these 41 genes have a free energy below −10 kcal/mol, which is significantly more genes than expected by chance (*p =* 4 × 10^−6^, Fisher's exact test).

The three most significant GO categories among the genes with weakly folded 5′-UTRs were related to Ty element retrotransposons (see Table 2). Ty element retrotransposons are stretches of DNA that replicate and move in the genome through RNA intermediates [38]. The Ty elements contain various genes in their sequences, e.g., proteases, integrases, and reverse transcriptases. The fact that they have weakly folded 5′-UTRs suggests that folding of their RNA is detrimental to their function or integration in the genome. Interestingly, Ty elements showed up in a study of RNA half-life where different methods of transcriptional inhibition were compared [39]. The RNA transcripts whose stability differed most between rpb1–1 inhibition on the one hand and Thiolutin, 1,10-phenanthroline, and 6-azauracil on the other hand were predominantly Ty elements. It may be worth investigating whether there is a connection between this difference in transcript stability and the lack of 5′-UTR secondary structure.

We found that 5′-UTR folding free energy was significantly positively correlated with both translational activity and mRNA half-life (see Table 1). These correlations were still significant after correction for GC-content, indicating that the correlations are not simply a secondary effect caused by nucleotide frequencies. Parker and colleagues showed that the insertion of secondary structures into the 5′-UTR of *PGK1* yeast mRNA inhibited translation and stimulated decay of *PGK1* [13]. Together, these findings suggest a widespread use of 5′-UTR secondary structure in post-transcriptional regulation. Our correlations may not be caused by any biochemical mechanism, e.g., transcripts of one evolutionary origin could have both strongly folded 5′-UTRs and low translation rates, whereas transcripts of another evolutionary origin could have weakly folded 5′-UTRs and high translation rates. Nevertheless, we believe that the correlations do reflect more direct connections. Our findings may be explained by an inhibitory effect of 5′-UTR secondary structure on translation initiation combined with competition between translation and decay. However, more direct biochemical pathways preferentially degrading mRNA with 5′-UTR secondary structure might also exist. Early support for the inhibitory effect of 5′-UTR secondary structure on translation came from insertion of hairpin loops into 5′-UTRs [9,10]. Later studies have shown connections between mRNA 5′ secondary structure and proteins important for translation such as eIF4A [15]. Competition between translation and decay has been proposed because both may require cap access [20,33]. Moreover, during translation the mRNA is circularized through interactions between cap-binding translation initiation factors and the poly(A)-binding protein (PABP). This conformation presumably protects mRNA from degradation by preventing access to both the cap and the poly(A) tail, suggesting that also the poly(A) tail is important for competition [17]. We expected that such competition would be more easily seen for short-lived transcripts because degradation takes up a larger part of their lives. Indeed, our global analysis revealed that transcript half-life is positively correlated with both ribosome density and ribosome occupancy, in particular for short-lived transcripts (see Figure 5).

A major mediator of heat shock response is mRNA decay [40], and the mRNA decay profile is similar to the heat response [39]. In line with these observations, we found a positive correlation between 5′-UTR free energy and mRNA response to heat shock (Table 1), i.e., transcripts with weakly folded 5′-UTRs are, in addition to being relatively long-lived, relatively upregulated after a heat shock. Given that transcripts that are upregulated by heat shock have weakly folded 5′-UTRs, it is expected that they would be translated at relatively high rates. Indeed, the correlation between ribosome occupancy and relative upregulation 10 min after heat shock was 0.23 (*p <* 2 × 10^−60^; similar for 5 min). Of interest, the heat shock mRNA *Hsp90* in *Drosophila* has extensive secondary structure in its 5′-UTR. *Hsp90* translation is inefficient at normal growth temperature, and is activated by heat shock, perhaps by thermal destabilization of the secondary structure in the 5′-UTR [41]. It may be worthwhile to perform genome-wide protein abundance experiments of heat shock response to investigate whether preferential heat shock translation is a common mechanism.

We assessed whether transcripts associated with RBPs, or with sequence motifs associated with these RBPs in their 3′-UTRs, were over- or underrepresented among fast decaying transcripts or among transcripts with strongly folded 5′-UTRs. Puf proteins are known to enhance mRNA turnover or repress translation [42]. We found targets of Puf3p, Puf4p, and Puf5p proteins to be significantly associated with fast decay, extending an earlier study [43]. Perhaps of interest, we note that the three Puf proteins for which Gerber et al. identified sequence motifs [7] were associated with fast decaying transcripts, while the remaining two Puf proteins, as well as Mex67 and Yra1, instead tended to be associated with weakly folded 5′-UTRs.

To summarize, we found that (i) 5′-UTRs have higher folding free energies than other genomic regions and than expected from their nucleotide composition, (ii) secondary structures in 5′-UTRs likely play a role in mRNA translation and turnover on a genomic scale, and (iii) genes with strongly folded 5′-UTRs are generally rarer, harder to find experimentally, and less annotated. It is important to keep in mind that the highly significant correlations we have found are small, showing that folding of 5′-UTRs is, as expected, only one aspect of post-transcriptional regulation. However, the correlations may be larger in subgroups of mRNAs, such as mRNAs targeted by individual decay pathways [44] and specific RBPs [45]. An example of a larger correlation in a subgroup is our observation that translational activity and mRNA decay are highly correlated for mRNAs with short half-lives.

## Materials and Methods

### Untranslated regions.

The exact 5′- and 3′-UTR lengths are unknown for most yeast genes. Mignone et al. [1] estimated the average lengths for yeast as 134 nt for 5′-UTRs and 237 nt for 3′-UTRs. With these numbers in mind, we retrieved 50, 100, and 200 nt of predicted 5′-UTRs and 237 nt of predicted 3′-UTRs from SGD for the 5,888 ORFs annotated as verified in SGD. As three additional control groups of 50-nt sequences, we retrieved nt 4–53 downstream (the first 50 nt following the start codon) for each of the 5,888 ORFs, the first 50 nt of the 3′-UTR region for each of the 5,888 ORFs, and 5,888 randomly chosen 50-nt sequences from intergenic regions from SGD.

### Folding of RNA secondary structures.

We used the RNAfold program in the Vienna RNA package [25] with default values for parameters (*T* = 37 °C) to compute secondary structures from RNA sequences. For each sequence, we used the free energy of the minimum free energy structure (the most negative Δ*G*) as a measure for secondary structure formation. For a given sequence, there may be other structures with similar Δ*G,* but we are interested in the possible free energy change in folding and not the secondary structure itself. A low Δ*G* corresponds to a strongly folded UTR, while a high Δ*G* corresponds to a weakly folded UTR. To avoid any pitfalls with using the free energy of the most strongly folded structure for each sequence, we also performed our analysis using the ensemble free energies [25], and none of the conclusions presented in this study changed. In fact, the correlations were typically somewhat more significant using ensemble averages. The free energies for all 5,888 genes are available in Dataset S1.

### Transcript feature datasets.

Translation profiles measured by Arava et al. [30] were downloaded from http://genome-www.stanford.edu/yeast_translation/. From this dataset, the number of bound ribosomes, the ribosome density (number of ribosomes per unit ORF length), and the ribosome occupancy (the fraction of the transcripts engaged in translation) were extracted for 5,700 genes, together with the mRNA copy number for 5,643 genes. Half-lives for 4,687 genes measured by Wang et al. [32] were downloaded from http://genome-www.stanford.edu/turnover/. A second dataset of mRNA half-lives for 6,092 genes [39] was obtained from http://hugheslab.med.utoronto.ca/Grigull/. For this dataset, the decay ratios 5 min after temperature shift of the rpb1–1 strain were used. Changes in transcript abundance in cells responding to heat shock for 5,259 genes measured by Gasch et al. [46] were downloaded from http://www-genome.stanford.edu/yeast_stress/. A dataset containing protein abundance information for 2,044 genes, constructed by Greenbaum et al. [47] by merging publicly available two-dimensional electrophoresis and MudPit data, was downloaded from http://bioinfo.mbb.yale.edu/expression/prot-v-mrna. Another dataset containing protein abundance information for 1,669 genes was obtained from the experiment by Ghaemmaghami et al. [48]. A merger of these two protein abundance datasets was obtained from Beyer et al. [31], along with a set of ribosome densities normalized by transcript length instead of ORF length.

### Dinucleotide shuffling.

Each native 5′-UTR sequence was shuffled 100 times keeping the dinucleotide frequencies constant using a publicly available implementation [28] of an algorithm developed by Altschul and Erickson [29]. For each 5′-UTR, the mean and the SD of the free energies of its randomized sequences were calculated. A *Z*-score was defined for each 5′-UTR as the free energy of the native sequence minus the mean of its randomized sequences divided by the SD of its randomized sequences [49].

### Statistical analysis.

Pearson correlations, Spearman rank correlations, Fisher's exact tests on 2 × 2 contingency tables, Mann–Whitney *U* tests, *t*-tests, and corresponding *p*-values were calculated using the statistics package R [50]. For Pearson correlations, *p*-values were calculated as the probability of obtaining a better correlation by chance if the two vectors were drawn independently from a Gaussian distribution. The multivariate linear model was done in R as well, and *p*-values were obtained with the ANOVA test of a linear model. All *p*-values were two-sided.

### GO analysis.

The 5,888 genes were mapped to 3,678 GO categories [34] using annotations from SGD. The genes were ranked according to Δ*G* in both increasing and decreasing order, separately, and a Wilcoxon rank sum test was employed for each GO category using Catmap [35]. Catmap outputs *p*-values calculated as the probability that a random ordering of the genes produces a lower, or equally low, Wilcoxon rank sum as the ordering investigated. The *p*-values were multiplied with the number of categories (3,678) to obtain Bonferroni corrected *p*-values.

## Supporting Information

Dataset S1 Calculated Folding Free Energies for All 5,888 Genes (487 KB TXT)

## Abbreviations

GO: Gene Ontology
nt: nucleotide(s)
ORF: open reading frame
RBP: RNA-binding protein
SD: standard deviation
SEM: standard error of the mean
SGD: *Saccharomyces* Genome Database
UTR: untranslated region
